# Serum Procalcitonin, Hematology Parameters, and Cell Morphology in Multiple Clinical Conditions and Sepsis

**DOI:** 10.1002/jcla.25100

**Published:** 2024-09-21

**Authors:** Pingfeng Feng, Yongjian He, Ping Guan, Chaohui Duan, Junjie Huang, Zhixin Chai, Jingjing Wang, Huifei Zheng, Junxu Luo, Yuhuan Shi, Xin Li, Huayi Huang

**Affiliations:** ^1^ Department of Laboratory Medicine Nanfang Hospital Affiliated to Southern Medical University Guangzhou Guangdong China; ^2^ Department of Laboratory Medicine Guangzhou Thoracic Hospital Guangzhou Guangdong China; ^3^ Department of Laboratory Medicine Sun Yat‐Sen Memorial Hospital of Sun Yat‐Sen University Guangzhou Guangdong China; ^4^ Division of the In Vitro Diagnostics Mindray Corporation Shenzhen Guangdong China; ^5^ Division of the In Vitro Diagnostics Mindray North America Mahwah New Jersey USA; ^6^ Guangzhou Daan Gene Corporation Guangzhou Guangdong China; ^7^ Department of Surgical Oncology Roswell Park Comprehensive Cancer Center Buffalo New York USA

**Keywords:** C‐reactive protein (CRP), digital automated cell morphology, neutrophil toxicity, procalcitonin (PCT)

## Abstract

**Background:**

The clinical value of procalcitonin (PCT) in infection diagnosis and antibiotic stewardship is still unclear. This study aimed to investigate the association between serum PCT and different clinical conditions as well as other infectious/inflammatory parameters in different septic patients in order to elucidate the value of PCT detection in infection management.

**Methods:**

Chemiluminescence immunoassay was used for serum PCT analysis. Hematology analysis was used for complete blood cell count. Digital automated cell morphology analysis was used for blood cell morphology examination. Blood, urine, and stool cultures were performed according to routine clinical laboratory standard operating procedures. C‐reactive protein (CRP) was analyzed by immunoturbidimetry. Erythrocyte sedimentation rate test was performed using natural sedimentation methods.

**Results:**

Outpatients, ICU patients, and patients under 2 years of age with respiratory infections had higher serum PCT levels. Septic patients had the highest‐serum PCT levels and other infection indexes. PCT levels in the blood, urine, and stool culture‐positive patients were significantly higher than in culture‐negative patients. The neutrophil granulation and reactive lymphocytes were observed together with the PCT‐level increments in different septic patients, and these alterations were lessened after treatment. There was no significant change in monocyte morphology between pre‐ and posttreatment septic patients.

**Conclusions:**

Serum PCT is associated with neutrophil cytotoxicity and lymphocyte morphology changes in sepsis; thus, the combination of neutrophil and lymphocyte digital cell morphology evaluations with PCT detection may be a useful examination for guiding the clinical management of sepsis.

## Graphical abstract

Serum procalcitonin (PCT), hematology parameters, and cell morphology in different clinical conditions and sepsis; the association of serum PCT levels and neutrophil cytotoxicity and lymphocyte morphology changes using the digital automated cell morphology technology and other inflammatory/infection parameters.

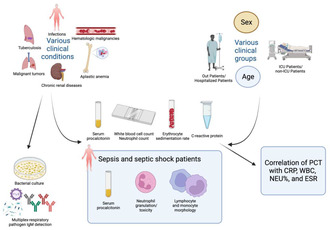

## 1 Introduction

Procalcitonin (PCT) is a 116‐amino‐acid residue first identified and described by Le Moullec et al. [1, 2], and its diagnostic significance was not recognized until 1993. Since then, numerous studies have found that elevated serum PCT levels were associated with bacterial infections but not associated with viral infections [2]. However, when compared with other inflammatory markers such as C‐reactive protein (CRP), PCT was not better as a marker of bacterial infection in emergency patients [3]. For clinical considerations, serum PCT levels decreased upon antibiotic treatment of bacterial infection [4], and a shorter duration of antibiotic therapy was achieved when PCT monitoring was used. PCT‐guided antibiotic treatment in ICU patients with infection and sepsis resulted in improved survival rates and lower antibiotic treatment durations [5]. However, another study found that serum PCT was elevated in other conditions as well, including renal dysfunction and heart failure, and not only in bacterial infection, thus making it difficult to interpret PCT levels [6]. Although the U.S. Food and Drug Administration (FDA) approved the use of PCT to guide antibiotic treatment in lower respiratory tract infections and sepsis years ago, the use of PCT‐guided antibiotic initiation is still controversial [7]. For instance, in pneumonia diagnosis, the diagnostic accuracy of PCT was only moderate and lower than that of interleukin 6 and CRP [8]. The clinical value of PCT was lower than expected. Furthermore, PCT was significantly elevated in severe and critical Coronavirus Disease 2019 (COVID‐19) patients, and it is unclear whether this elevation was because of a severe immune reaction to the severe acute respiratory syndrome coronavirus 2 (SARS‐CoV‐2) virus or subsequent bacterial infection [9, 10, 11]. Another concern is that the usefulness of PCT and other inflammatory markers, such as CRP and interleukin 6, as biomarkers for infection has long been debated because of their low specificity and sensitivity [12]. It is believed that the efficacy of PCT in differentiating infectious from noninfectious forms of systemic inflammatory response syndrome and in stratifying morbidity and mortality risk is limited. That said, PCT has been considered a useful tool for diagnosing late‐onset sepsis, bacterial meningitis, and other forms of organ‐related bacterial infections; thus, it can be used for guiding antibiotic treatments in critical patients [13].

As mentioned above, although PCT is widely used in the evaluation of various infections, contradictory and uncertain reports still exist. We hypothesized that it would be interesting to observe serum PCT levels in different clinical conditions with associations to other laboratory parameters. These parameters included reactivities of immune cells such as neutrophils, lymphocytes, and monocytes in peripheral blood and the changes in these reactions pre‐ and posttreatment in different septic patients as evaluated via digital automated cell morphology technology. Herein, we report the serum PCT levels in different clinical conditions, concentration changes pre‐ and posttreatment, and observed associations between serum PCT levels and the reactions of neutrophils, lymphocytes, and monocytes in different septic patients. We hope that this will contribute to a better understanding of the value of PCT detection in disease management.

## 2 Methods

### 2.1 Project Design

This study was designed as a prospective and observational multicenter study. The intended study period was from February 2020 to February 2021; however, it was instead conducted from February 2020 to August 2021. Various treatments may have impacted serum PCT levels in hospitalized patients. Furthermore, the half‐life of PCT in the bloodstream is short (20–24 h); thus, patients were grouped as outpatient or hospitalized. For example, (1) outpatient with infection group: these patients were usually in an urgent hospital visit without antibiotics use. In this group, patients who came to various clinics for care due to fever were subjected to the order of complete cell count (hematology analysis), procalcitonin assay, C‐reactive protein assay, and other laboratory tests based on the physician's judgment; patients were also inquired for any known use of antibiotics prior to the visit. Patients were either admitted for hospitalization and further treatments or sent home. (2) Hospitalized patients with infection group: this group consisted of patients hospitalized in the ICU, respiratory medicine, and other departments. These patients were usually treated with various medications including antibiotics and other procedures upon infection. We were unable to group patients based on the specific bacterium, type of inflammation, or virus infection present. Further raw grouping was done based on the types of diseases and organs involved as well as age. Exclusions include (1) patients with ambiguous diagnoses; (2) patients with known antibiotic use after infection and before enrollment; and (3) patients who died prior to conclusion of observation. Patients in the emergency department and outpatient clinics were diagnosed by physicians of the respective departments. The diagnosis was made following the guidelines for different diseases. The blood sample was drawn for hematology and chemistry analysis before treatment. For hospitalized patients, the final diagnosis was determined based on their diagnosis at discharge. Only patients with clear diagnoses were enrolled in the study, and only the first three diagnoses were used for patients who had multiple diagnoses at discharge.

### 2.2 Participating Hospitals and Ethical Approval

Three Class A tertiary hospitals in southern China participated in this study. Nanfang Hospital is a general hospital affiliated with Southern Medical University and admits patients with different types of diseases; thus, patients with infections are more common there. Sun Yat‐Sen Memorial Hospital is also a general hospital affiliated with Sun Yat‐Sen University; however, it focuses on malignant diseases, so patients with neoplasms are more common there. Guangzhou Thoracic Hospital specializes in treating thoracic diseases, including tuberculosis, thoracic cancers, and orthopedic diseases. This study was approved by the Institutional Review Board/Ethics Committee of Nanfang Hospital of Southern Medical University, Guangzhou Thoracic Hospital, and Sun Yat‐Sen Memorial Hospital of Sun Yat‐Sen University (NFEC‐2020‐235, 2020 (No. 63) and SKSEC‐KY‐KS‐2022‐149, respectively).

### 2.3 Testing Parameters and Results Collection

The blood for hematology and chemistry analysis was normally taken on the day following admission. If a patient had several test results, only the first was taken for statistical analysis. Blood, urine, and stool cultures were made based on the treatment requirements of the patients. In total, 1193 patients were eligible for data analysis, with ages from 1 day after birth–93 years old and gender breakdowns of 805 males and 388 females. Among the cohort, 34 cases were infants under 2 years of age (1 day after birth–2 years). Detailed information is shown in Table S1 Patient Demographics. Serum PCT, complete blood cell count (for septic patients, automatic digital morphology examination was performed), CRP, and erythrocyte sedimentation (ESR) results were collected. Blood and urine culture results were collected when available, and ambiguous results or failures in culture due to poor sampling were excluded.

### 2.4 Chemiluminescence Immunoassay of Procalcitonin

Fasting venous blood was drawn in the morning on the day following hospitalization or at the outpatient clinic visit. The samples were sent to the clinical laboratory within 30 min of sampling and processed following the laboratory standard operating procedures. Serum was isolated, and PCT was analyzed on the Roche e801 (Roche Diagnostics, Indianapolis, Indiana, USA) with the Elecsys BRAHMS PCT reagent kit, the Mindray CL‐6000i (Mindray Corporation, Shenzhen, Guangdong, China) (for samples from Nanfang Hospital) with the Procalcitonin (CLIA) reagent kit, or the VIDAS B.R.A.H.M.S. PCT (Biomerieux, Marcy L'Etoile, France) (for samples from Guangzhou Thoracic Hospital and Sun Yat‐Sen Memorial Hospital) chemiluminescence immunoassay platforms following the manufacturers' recommendations. Results from the three different platforms were comparable.

### 2.5 Culture of Pathogens

Blood cultures were performed using a BD BACTEC FX blood culture system (Becton‐Dickinson, Franklin Lakes, New Jersey, USA) for samples from Nanfang Hospital and Guangzhou Thoracic Hospital and a BACT/ALERT blood culture system (Biomerieux, Marcy L'Etoile, France) for samples from Sun Yat‐Sen Memorial Hospital. Urine and stool cultures were performed following the laboratory standard operating procedures.

### 2.6 Hematology Analysis and Morphology Examination

Hematology analyses were performed for patient samples from Nanfang Hospital on a Sysmex XN9000 (Sysmex Corporation, Kobe, Japan), and a CellaVision DI60 digital morphology (CellaVision, Lund, Sweden) was used for morphology analyses following clinical laboratory standard operating procedures and the manufacturer's instructions.

### 2.7 C‐Reactive Protein Analysis

C‐reactive protein (CRP) analysis was performed for patient samples from Nanfang Hospital on a Roche C8000 modular clinical chemistry analyzer (Roche Diagnostics, Indianapolis, Indiana, USA) using an immunoturbidimetry‐based method following the manufacturer's instructions.

### 2.8 Erythrocyte Sedimentation Rate Test

The erythrocyte sedimentation rate (ESR) was measured on a Precil XC40 ESR analyzer (Precil Instruments Corporation, Beijing, China) for samples from Nanfang Hospital utilizing a natural sedimentation‐based method following the manufacturer's instructions.

### 2.9 Multiplex Respiratory Pathogen IgM Testing

An indirect immunofluorescent assay‐based multiplex respiratory pathogen IgM test kit (VIRCELL, Granada, Spain) was used to detect *Legionella pneumophila*, *Mycoplasma pneumoniae*, *Coxiella burnetii*, adenovirus, respiratory syncytial virus, influenza A virus, influenza B virus, and parainfluenza virus 1, 2, and 3 in the serum following the manufacturer's instructions.

### 2.10 Statistical Analysis

All data were analyzed using the SPSS version 26.0 statistical software (SPSS Inc., Chicago, Illinois, USA). Specifically, the Kruskal–Wallis test, Mann–Whitney *U* test, multiple comparisons, and Pearson correlation were used for analysis. A *p* < 0.05 was considered significant in difference. The statistical analysis was performed by a third‐party statistician. The original data were stored and are available upon request.

## 3 Results

### 3.1 Patient Demographics

Patient demographics in this study are shown in Table S1.

### 3.2 Serum PCT Levels in Different Clinical Conditions

**FIGURE 1:**
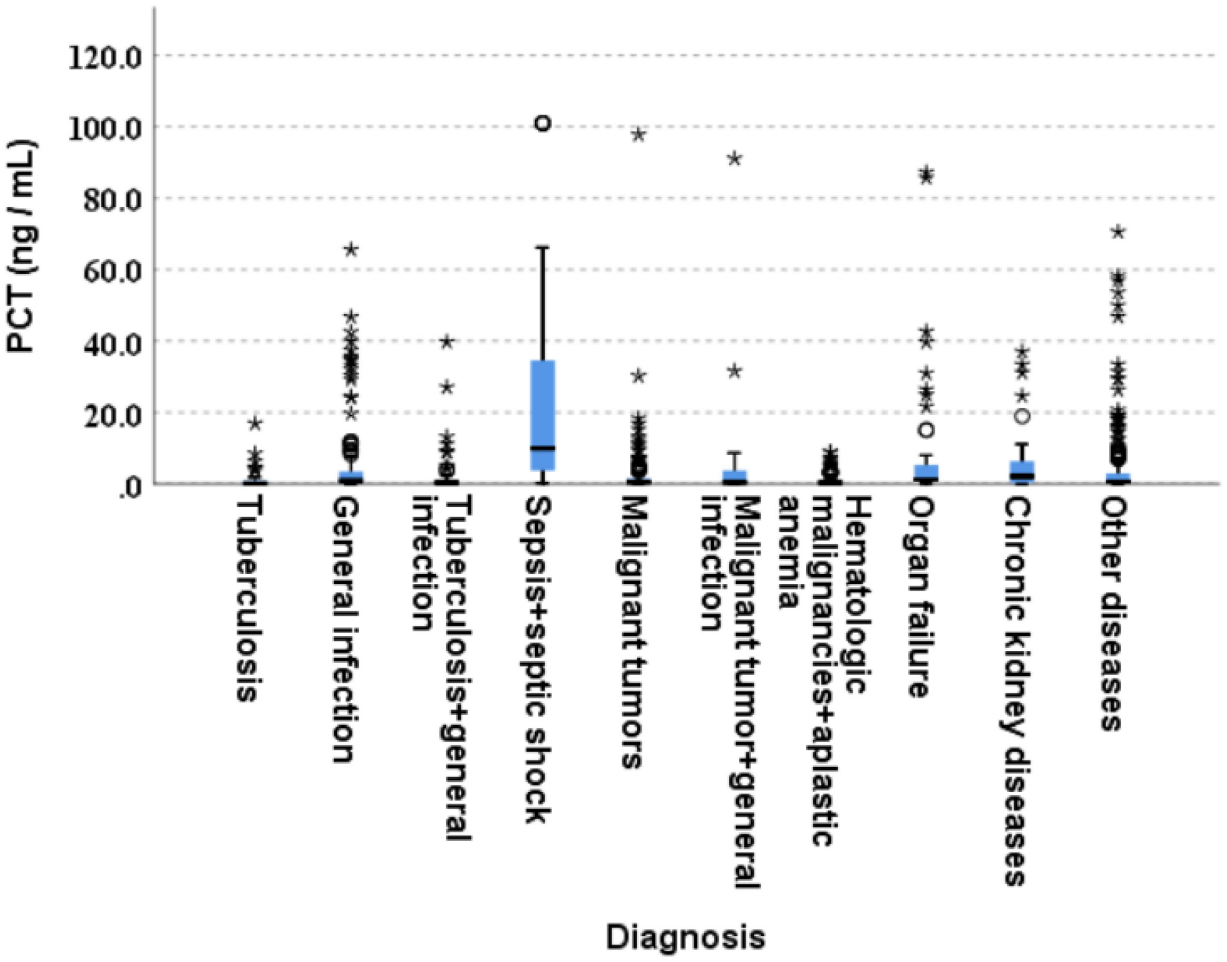
Shows the distribution of PCT concentration among different clinical conditions with sepsis + septic shock patient group presented the highest‐serum PCT levels.

Serum PCT levels were significantly different among different age groups (*p* < 0.05) with age ≤ 2 years being the highest; there was no significant difference in PCT levels between male and female patients (*p* = 0.245). PCT levels in the outpatient groups were significantly higher than in hospitalized patients (*p* = 0.000); patients in the intensive care unit (ICU) had higher PCT levels than non‐ICU patients (*p* = 0.000). Results also showed that patients from Nanfang Hospital had higher PCT levels than the other two hospitals (*p* = 0.000) (Table 1). Figure 1 shows that PCT levels among different clinical conditions were variable. There were significant differences in PCT levels among different clinical conditions, with sepsis and septic shock having had the highest (*p* = 0.000, Table 2), followed by chronic renal diseases, multiple organ failure, and common bacterial infections (upper respiratory tract infections, pyelonephritis, and enteric infections). The stepwise stepdown comparison of the average ranks of grouped conditions is shown in Table 3.

**TABLE 1:** Comparison of PCT levels in different clinical groups.

Factors		*n*	Median	P25–P75
Age range	≤ 2 years*	34	1.565	0.338–5.293
	2–50 years*	432	0.415	0.177–1.935
	> 50 years*	727	0.764	0.195–3.010
Kruskal–Wallis test	Statistics		13.744	
	*p* value (two‐tailed test)		0.001	
Sex	Male	805	0.721	0.200–2.960
	Female	388	0.451	0.176–2.970
Mann–Whitney *U* test	Statistics		−1.163	
	*p* value (two‐tailed test)		0.245	
Types of patients	Outpatient	166	3.930	1.378–7.260
	Hospitalized	1027	0.400	0.175–1.640
Mann–Whitney *U* test	Statistics		−12.687	
	*p* value (two‐tailed test)		0.000	
ICU patients or not	No	983	0.431	0.180–1.840
	Yes	210	1.965	0.655–7.208
Mann–Whitney *U* test	Statistics		8.459	
	*p* value (two‐tailed test)		0.000	
Hospital	Guangzhou Thoracic Hospital	203	0.410	0.050–1.710
	Nanfang Hospital*	787	0.729	0.237–3.080
	Sun‐Yat‐Sen Memorial Hospital	203	0.710	0.080–1.970
Kruskal–Wallis test	Statistics		31.393	
	*p* values (two‐tailed test)		0.000	

*Note:* *p < 0.05 compared with other featured groups.

**TABLE 2:** Comparisons of PCT levels of different clinical conditions (Kruskal–Wallis test).

Diagnosis	*n*	Median	P25–P75	Min–Max
Tuberculosis	57	0.130	0.040–0.845	< 0.05–16.880
Common bacterial infection + Tuberculosis	61	0.300	0.040–1.130	< 0.05–39.750
Hematology malignancies and aplastic anemia	112	0.306	0.176–1.248	< 0.05–8.930
Malignant tumors + Common bacterial infections	28	0.391	0.227–3.720	< 0.05–91.120
Malignant tumors	170	0.455	0.187–1.515	< 0.05–97.820
Other types of diseases	389	0.619	0.193–2.890	< 0.05–70.580
Common bacterial infections	239	0.881	0.222–3.510	< 0.05–598.000
Multiple organ failure	64	1.210	0.772–5.290	0.123–87.230
Chronic renal diseases	38	2.110	0.293–6.473	< 0.05–36.980
Sepsis and septic shock	35	9.940	3.660–39.070	0.199–340.000
Statistics		126.556		
*p* value (two‐tailed test)		0.000		

**TABLE 3:** Comparison of PCT levels among different clinical conditions (stepwise stepdown).

	Diagnosis^a^	Subset						
		1	2	3	4	5	6	7
Sample^b^	Tuberculosis	362.7						
	Common bacterial infections + Tuberculosis	448.2	448.2					
	Hematology malignancies and aplastic anemia		503.5	503.5				
	Malignant tumors			574.3	574.3			
	Other types of diseases			589.9	589.9	589.9		
	Malignant tumors + Common bacterial infections			605.6	605.6	605.6		
	Common bacterial infections				630.1	630.1		
	Chronic renal diseases					737.1	737.1	
	Multiple organ failure						766.9	
	Sepsis and septic shock							1030.7
Statistics		1.292	1.963	5.937	3.294	7.655	0.002	—^c^
*p* value (two‐tailed test)		0.256	0.161	0.115	0.348	0.054	0.967	—
Adjusted *p* value (two‐tailed test)		0.772	0.585	0.263	0.657	0.129	1.000	—

*Note:* Homogeneous subsets are based on asymptotic significances. The significance level is 0.05.

^a^ Grouping based on the PCT average rank.

^b^ Each cell shows the sample average rank of diagnosis.

^c^ Unable to compute because the subset contains only one sample.

### 3.3 WBC and Neutrophil Counts Among Different Clinical Conditions

Sepsis and septic shock patients had the highest WBC count in peripheral blood among all the different clinical conditions, followed by common bacterial infections, other types of diseases, and malignant tumors (Table S2). Similar to WBC, septic patients also had the highest NEU% among different clinical conditions, followed by multiple organ failure (Table S3).

### 3.4 Association Between Pathogen Culture and Multiplex Respiratory Pathogen IgM Test Results and PCT Levels

Results indicated that patients with positive urine/stool and blood cultures had higher serum PCT levels compared to those with negative cultures (*p* = 0.029 and 0.012, respectively); there was no significant difference in PCT levels between patients with positive and negative sputum cultures (*p* = 0.599). There was no association between multiplex respiratory pathogen IgM detection results and PCT levels (*p* = 0.706) (Table S4).

### 3.5 CRP Levels Among Different Clinical Conditions and the Correlation of PCT With CRP, WBC, NEU%, and ESR

The highest‐serum CRP levels were also observed in septic patients followed by common bacterial infection with tuberculosis, and the correlation among PCT, CRP, WBC, NEU%, and ESR was low (Pearson correlation *R* < 0.226) (Table S5).

### 3.6 Cytotoxicity/Granulation of Neutrophils in Septic Patients (Digital Automated Cell Morphology)

**FIGURE 2:**
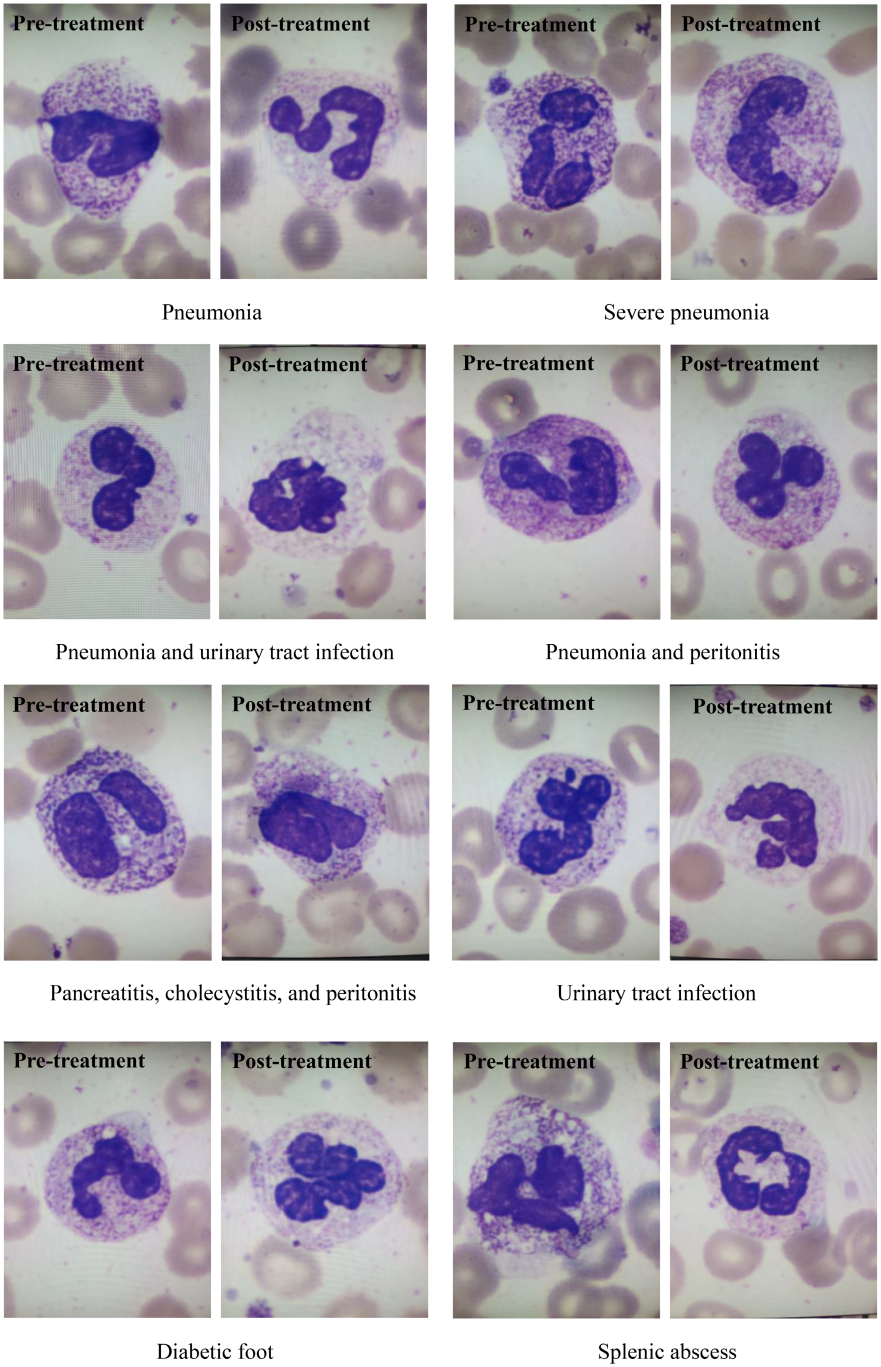
Shows that the granules (cytotoxicity) in the neutrophils are denser and coarser in septic patients before treatment. However, the granules were reduced after treatment (Wright–Giemsa staining, by automated digital morphology examination, magnification power: ×1000).

Results showed that the granules in the cytoplasm of neutrophils, which represented the cytotoxicity of the immune cells, were coarse and dense before treatment, but the granules were significantly reduced after treatment in different septic patients (Figure 2).

### 3.7 Morphology Changes of Lymphocytes in Septic Patients (Digital Automated Cell Morphology)

**FIGURE 3:**
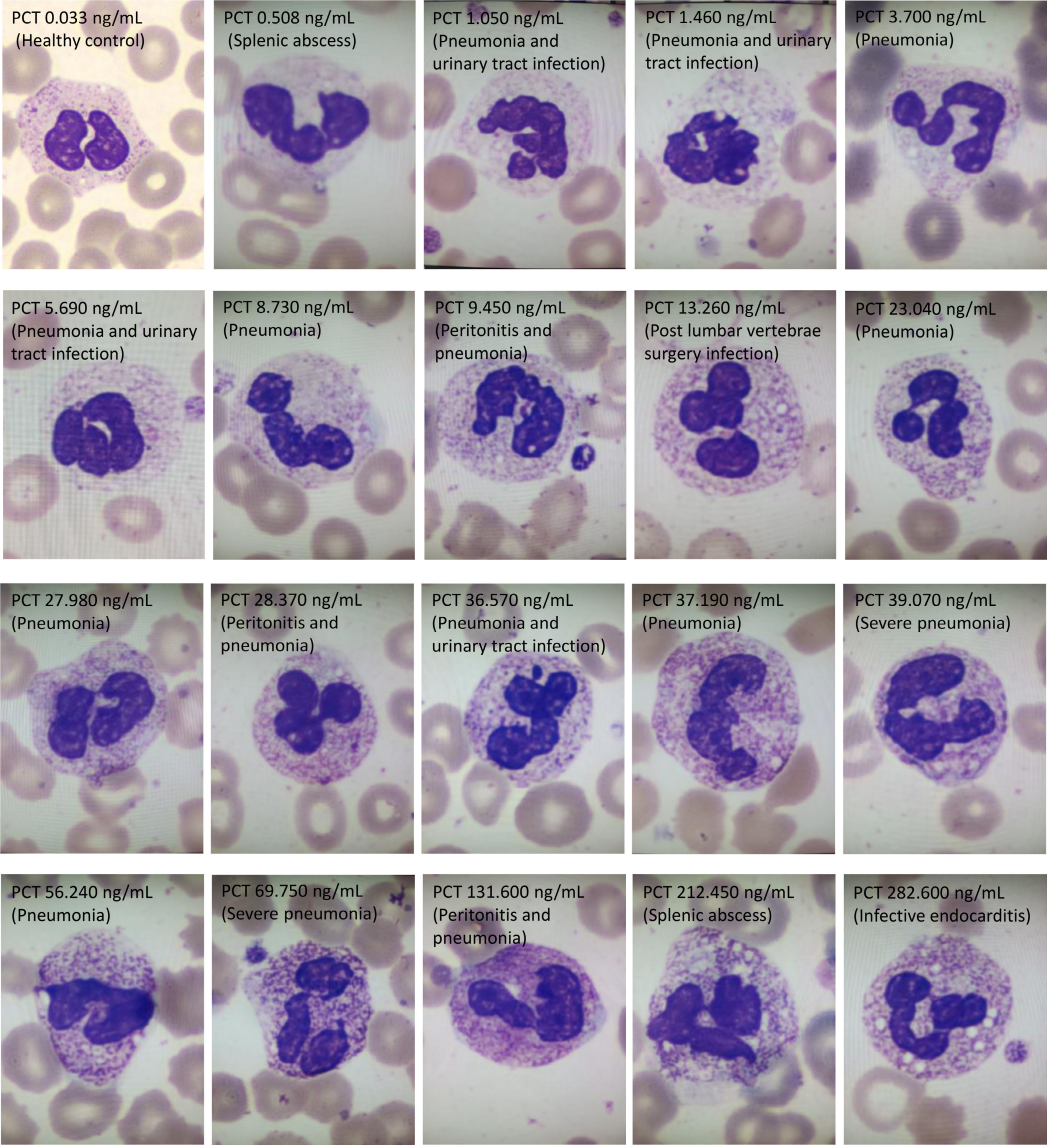
Shows that the granules in the neutrophils became denser and coarser upon the increments of PCT levels in different septic patients (Wright–Giemsa staining, by automated digital morphology examination, magnification power: ×1000).

There were more reactive lymphocytes and coarse cytoplasmic granules in septic patients before treatment; fewer reactive lymphocytes were seen after treatment (Figure 3).

### 3.8 Association Between Cytotoxicity of Neutrophils and PCT Levels

**FIGURE 4:**
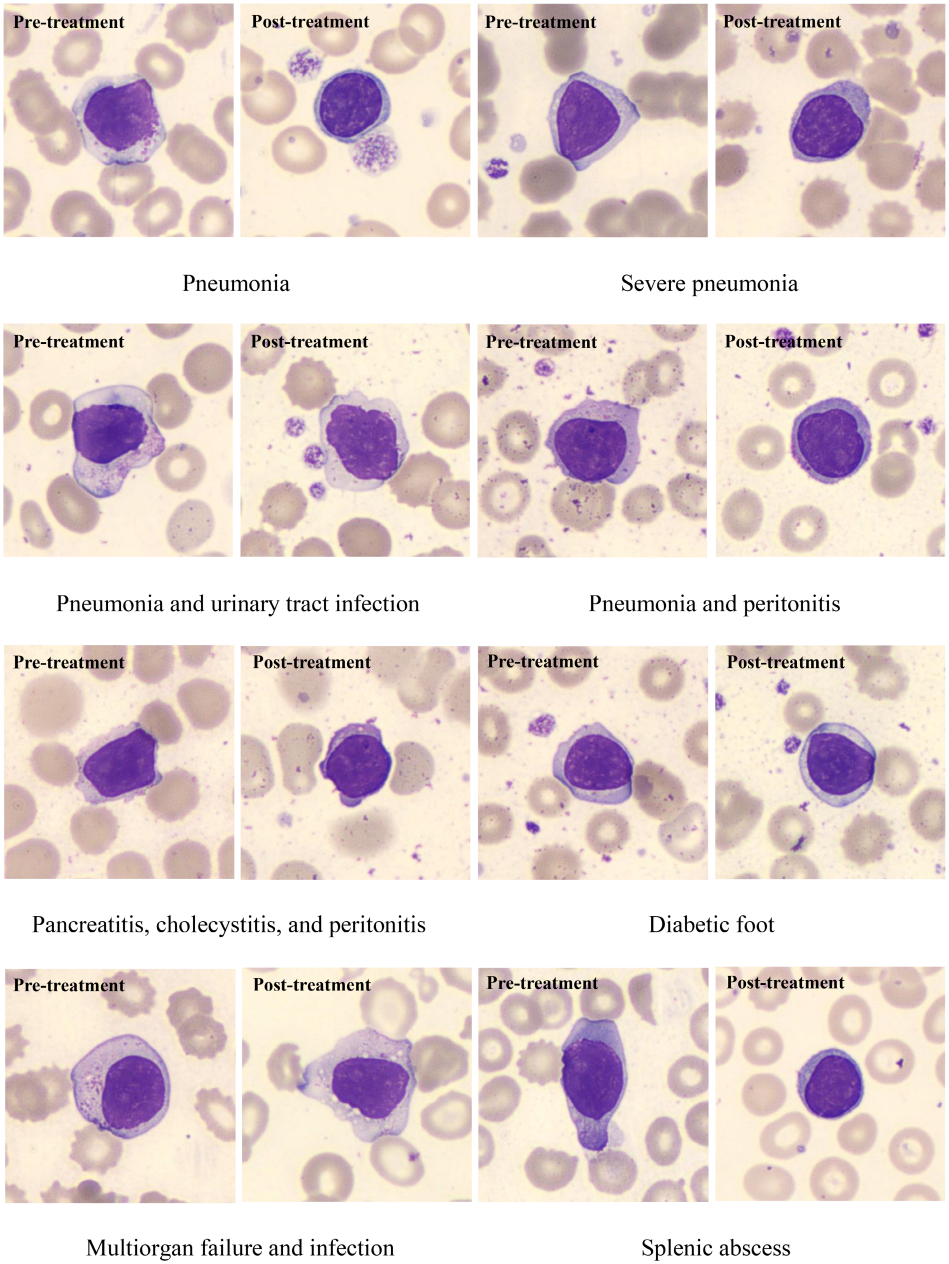
Shows that more reactive lymphocytes were seen in different septic patients before treatment (Wright–Giemsa staining, by automated digital morphology examination, magnification power: ×1000).

Fewer granules in the cytoplasm of neutrophils were found similar to those with serum PCT under approximately 5 ng/mL. The granules in the cytoplasm of neutrophils also became dense and coarse (Figure 4).

### 3.9 Examination Results of Morphology Changes of Monocytes in Septic Patients

It was found that the morphology of monocytes did not have a significant change between pre‐ and posttreatment in different septic patients (Figure S1).

## 4 Discussion

Although numerous studies indicated that PCT could be a helpful biomarker for characterizing bacterial and viral infections and thus guiding the use of antibiotics in clinical settings, the results were still ambiguous and controversial [14]. The capability of PCT in differentiating infection from inflammation was another issue [15]. The association between serum PCT level and the severity of infection has also not been fully elucidated [16]. In this framework, this study further detailed some factors and uncovered novel phenomena as described below:

Various serum PCT levels were observed in different age groups, with age ≤ 2 years having the highest levels. The reasons for this phenomenon may be complex, but it could be shown that most of the patients in this group had infant respiratory distress syndrome (IRDS), pneumonia, and other infections. Similarly, previous studies found that PCT was an ideal biomarker for infections including pneumonia and IRDS in neonates, infants, and children [17, 18, 19]. Our results further indicated the possibility of infection or IRDS in this age group as reported previously and the clinical usefulness of PCT testing. Interestingly, the PCT levels in the outpatient groups were significantly higher than in hospitalized patients, suggesting that outpatients with infection‐related manifestations usually arrived at the hospital for care without antibiotics treatment. Because PCT is a precursor of calcitonin, it is undetectable in healthy conditions; PCT is upregulated by cytokines released in response to inflammation, especially in bacterial infections. Serum PCT levels rise rapidly in response to the stimuli, and its peak levels correlate with the intensity of the stimuli. The half‐life of PCT is short (25–30 h), and levels decline rapidly with resolution of inflammation such as after antibiotics use [20]; hospitalized patients are usually treated with antibiotics or other therapies. Consistent with previous reports, patients in the ICU had higher PCT levels than non‐ICU patients [21, 22]. Therefore, PCT may be a helpful marker for guiding the use of antibiotics in ICU patients. As described above, patients from Nanfang Hospital had higher PCT levels than those from the other two hospitals (Table 1) because of the high volume of emergency patients who visited this hospital; it was followed by Sun Yat‐Sen Memorial Hospital, which was also a general hospital accepting emergency patients. Septic patients had the highest PCT levels (Figure 1 and Tables 2 and 3), followed by multiple organ failure patients, which was usually caused by severe infection or infections as comorbidity which subsequently led to septic shock and death. During the disease course, PCT levels and their responses to antibiotics use were associated with the septic shock severity and sepsis‐related organ failure assessment (SOFA) score [23, 24, 25]; in chronic renal diseases, the association was due to chronic inflammation rather than infection [26] (Tables 2 and 3). This phenomenon has also been observed in chronic liver diseases [27].

PCT has been correlated with other infection/inflammation markers such as CRP and WBC count. Serum PCT was found to correlate well with WBC count in septic patients in the emergency department, and the combination of PCT and WBC could be a reliable diagnostic and prognostic biomarker for sepsis [28]. Our results also showed that the highest WBC counts were observed in septic patients, followed by common bacterial infections; however, WBC also increased in other types of diseases including malignant tumors (Table S2). This could indicate potential infection status in those diseases or nonspecificity of PCT reaction to inflammation, which needs to be further investigated.

Similar to the WBC count, the NEU% was also highest in septic patients (Table S3). WBC count and NEU% are common indexes for bacterial infection in clinical practice.

As mentioned above, serum PCT level directly reflected the severity of infection. It was significantly higher in blood and urine/stool culture‐positive patients than in negative patients. There was no significant difference in PCT levels between positive and negative sputum cultures, and this could be interpreted as a nonspecific culture result from sputum, making the comparison insignificant. One study found an association between PCT levels and respiratory tract viral infections [29]. However, we did not observe an association between multiplex respiratory pathogen IgM detection results and PCT levels (Table S4). Thus, repeat test or surveillance both for culture and IgM detection may be needed to confirm the infection.

Although PCT, CRP, WBC, and NEU% were all elevated in septic patients, the Pearson correlation analysis results indicated that the correlation of PCT with these parameters and the ESR was low (Table S5). Similar results were found in burn patients by Barati et al. [30], which indicated WBC, neutrophil count, CRP, and ESR had little value as diagnostic indicators for sepsis in burn cases. Nonetheless, other studies found that a combination of PCT with WBC presented high diagnostic performance for patients who presented infection or sepsis in the emergency department [28, 31]. This suggests that the patients observed must have been those who have never been treated and with the same type of disease. However, one study found that serum PCT and CRP measured at admission in the pediatric intensive care unit revealed that in most cases, serum PCT was already very high at onset, and it did not increase significantly afterward or vary with the age of patients. Patients with septic shock or multiple organ dysfunction syndromes had higher‐serum PCT levels [32]. Altogether, the value of combinatory analysis of PCT, CRP, and NEU% in the evaluation of the severity of infections is still controversial.

Toxic granulation in the cytoplasm of neutrophils is usually associated with infection, and the density and size are also associated with the severity of infection. Thus, in this study, we wanted to observe if the toxic granulations in the neutrophils were associated with serum levels of PCT in septic patients as well as any changes upon treatment. Interestingly, we found that in septic patients, the cytotoxicity of neutrophils was different between pre‐ and posttreatment observations, and the granules in the cytoplasm of neutrophils were coarser and denser before treatment; however, the granules were significantly reduced after treatment (Figure 2). Our results indicated that PCT under approximately 5 ng/mL presented fewer granules in the cytoplasm of neutrophils, similar to that of healthy people (data not shown); as PCT level increased, the granules in the cytoplasm of neutrophils also became denser and coarser (Figure 3). The underlying mechanism of this toxicity is unclear, and some studies suggested that an inflammation pathway including the IL‐6, iNOS, TNF‐α, and CD64 was involved [33, 34], and a set of genes and proteins were found to play a role in neutrophil granulopoiesis in sepsis [35]. Chivukula et al. reported a septic case with *Klebsiella pneumoniae* resistant to carbapenems in which the WBC and neutrophil counts were variable with elevated serum PCT and toxic granule appearance in neutrophils. However, the toxic granules disappeared after treatment in conjunction with the decline in PCT levels [36]. Thus, the morphology changes associated with PCT in septic patients could be a useful marker in evaluating therapeutic responses and prognosis. By comparing the report from Chivukula et al. with a report on one case with continuous monitoring, our study observed 35 cases of septic patients with different infection sites and various PCT levels associated with toxic granules. However, we were unable to characterize the specific pathogen (bacterium) in each patient. This demerit would be resolved in future studies.

Reactive lymphocytes could be seen in bacterial and viral infections. Similar to the toxicity/granulation of neutrophils, we also observed that there were more reactive lymphocytes and coarser cytoplasmic granules in pretreatment compared to posttreatment for different septic patients (Figure 4). The underlying mechanism is unclear, and we were unable to characterize the subset of lymphocytes responsible for this phenomenon. Results might suggest a “stress” status under chronic infection or immune stimulation, since the reactive lymphocytes are usually CD8+ T subset [37, 38] which are triggered by the immune response against the infection and inflammation [39, 40]. Study found that cytokines such as CCL2/MCP‐1, IL‐10, IL‐2, and TNF‐alpha were associated with lymphocyte morphology changes and other laboratory test results related to inflammation in sepsis, and those parameters were decreased in sepsis recovery [41]. The detailed mechanism needs to be further investigated.

Morphologic changes in monocytes can be seen in certain viral infections. Thus, we wanted to see if this phenomenon occurred in sepsis. We did not observe significant morphology changes in monocytes between pre‐ and posttreatment results in different septic patients (Figure S1).

A study found that PCT levels were significantly different between septic patients with different culture results. The cut‐off for culture‐negative patients was 1.3 ng/mL while the cut‐off for culture positive was 2.20 ng/mL [42]; this suggests that the level of PCT was consistent with the positivity of culture.

## 5 Conclusions

This study has the following conclusions: Patients with infections such as pneumonia showed high‐serum PCT levels, and septic patients possessed the highest‐serum PCT levels. However, high‐serum PCT levels were also observed in patients with multiple organ failure, chronic renal diseases, and malignancies. PCT, WBC, and NEU% were increased in septic patients, but the correlations between PCT and WBC, NEU%, and ESR were low. This indicates that these parameters may not increase simultaneously in severely infected patients; patients with positive blood and urine/stool cultures had higher‐serum PCT levels than those with negative cultures. There was an association between the cytotoxic presentation of neutrophils and the reactive phenotype of lymphocytes and PCT levels but not monocytes in septic patients—a result that merits further investigation. Furthermore, the granulation presentations of neutrophils in septic patients were reduced after treatment. Another strength of this study is that both outpatients and hospitalized patients with different clinical conditions were observed; this provided broadened information regarding the clinical values of PCT, as previous works observed either outpatients or hospitalized patients only.

## 6 Limitations of This Study

This study contained the following limitations: (1) We were unable to group the patients into outpatients and hospitalized patients for more specific analysis; (2) It was difficult to identify whether hospitalized patients were treated with or had used any antibiotics while taking the blood sample for PCT analysis, especially for patients in the ICU unit due to their complex conditions; (3) Most of the patients observed in this study did not have a culture result due to failure of adherence or inappropriate sampling for culture. For those with a culture result, we were unable to analyze the associations between serum PCT levels and the specific bacterial strains due to insufficient sample size; (4) This study was unable to categorize the patients into more detailed groups due to insufficient patient cohort size; (5) We were unable to further study the detailed mechanisms of the toxicity of neutrophils and morphology changes of lymphocytes in relation to PCT levels in septic patients; and (6) Diagnosis was made based on the departments where patients were admitted, so the diagnosis was department prioritized and might have been biased. Furthermore, a patient may have had multiple diagnosis when discharged. Future work will focus on the issues mentioned above.

## Author Contributions

P.F., Y.H., J.H., Z.C., and Y.S. were involved in the investigation and data collection; P.G., C.D., J.W., H.Z., and J.L. were project organizers; X.L. is the principal investigator; H.H. designed the project and wrote the manuscript, as a co‐principal investigator.

## Conflicts of Interest

The authors declare no conflicts of interest.

## Supporting information


Figure S1.

Tables S1–S5.

## Data Availability

All data in this study on which the conclusions of the manuscript rely are available upon request from the corresponding authors for researchers who meet the criteria.
