# Supplementary material for: Serum Procalcitonin, Hematology Parameters, and Cell Morphology in Multiple Clinical Conditions and Sepsis
Source: J Clin Lab Anal. 2024 Sep 21;38(19-20):e25100. doi: 10.1002/jcla.25100 (PMC11520939; doi:10.1002/jcla.25100)
Supplement: Supplementary file 1 — Figure S1. [file JCLA-38-e25100-s002.docx]

**Figure S1 Morphology examination of monocytes of different septic patients pre- and post-treatment**

Pneumonia, peritonitis Splenic abscess

Pneumonia, urinary tract infection Diabetic foot

Severe pancreatitis, acute peritonitis Severe pneumonia

Multiple organ failure

**Figure legends**

**Fig. S1**

Morphology examination of monocytes of different septic patients pre- and post-treatment. Results indicated that there were no morphological changes in monocytes in different septic patients before and after treatment (Wright-Giemsa staining, by automated digital morphology examination, magnification power: ×1000).
