# Supplementary material for: Serum Procalcitonin, Hematology Parameters, and Cell Morphology in Multiple Clinical Conditions and Sepsis
Source: J Clin Lab Anal. 2024 Sep 21;38(19-20):e25100. doi: 10.1002/jcla.25100 (PMC11520939; doi:10.1002/jcla.25100)
Supplement: Supplementary file 2 — Tables S1–S5. [file JCLA-38-e25100-s001.docx]

**Table S1 Patient demographics**

| Diagnosis | Age (year) |  |  | Sex |  |
| --- | --- | --- | --- | --- | --- |
|  | Median (Min-Max) | category | n (%) | category | n (%) |
| Tuberculosis | 54.0 (16.0 - 89.0) | ≤2 | 0 (0%) | Male | 45 (78.9%) |
|  |  | 2-50 | 21 (36.8%) | Female | 12 (21.1%) |
|  |  | >50 | 36 (63.2%) |  |  |
|  |  |  |  |  |  |
| General infection | 57.0 (0.0 - 92.0) | ≤2 | 8 (3.3%) | Male | 172 (72.0%) |
|  |  | 2-50 | 84 (35.1%) | Female | 67 (28.0%) |
|  |  | >50 | 147 (61.5%) |  |  |
|  |  |  |  |  |  |
| Tuberculosis + general infection | 56.0 (18.0 - 87.0) | ≤2 | 0 (0%) | Male | 48 (78.7%) |
|  |  | 2-50 | 24 (39.3%) | Female | 13 (21.3%) |
|  |  | >50 | 37 (60.7%) |  |  |
|  |  |  |  |  |  |
| Sepsis + septic shock | 56.0 (9.0 - 87.0) | ≤2 | 0 (0%) | Male | 22 (62.9%) |
|  |  | 2-50 | 11 (31.4%) | Female | 13 (37.1%) |
|  |  | >50 | 24 (68.6%) |  |  |
|  |  |  |  |  |  |
| Malignant tumors | 59.0 (6.0 - 84.0) | ≤2 | 0 (0%) | Male | 113 (66.5%) |
|  |  | 2-50 | 39 (22.9%) | Female | 57 (33.5%) |
|  |  | >50 | 131 (77.1%) |  |  |
|  |  |  |  |  |  |
| Malignant tumor + general infection | 56.5 (38.0 - 84.0) | ≤2 | 0 (0%) | Male | 21 (75.0%) |
|  |  | 2-50 | 10 (35.7%) | Female | 7 (25.0%) |
|  |  | >50 | 18 (64.3%) |  |  |
|  |  |  |  |  |  |
| Hematologic malignancies + aplastic anemia | 33.5 (0.7 - 85.0) | ≤2 | 8 (7.1%) | Male | 67 (59.8%) |
|  |  | 2-50 | 73 (65.2%) | Female | 45 (40.2%) |
|  |  | >50 | 31 (27.7%) |  |  |
|  |  |  |  |  |  |
| Multiple organ failure | 60.0 (17.0 - 92.0) | ≤2 | 0 (0%) | Male | 43 (67.2%) |
|  |  | 2-50 | 21 (32.8%) | Female | 21 (32.8%) |
|  |  | >50 | 43 (67.2%) |  |  |
|  |  |  |  |  |  |
| Chronic kidney diseases | 61.0 (1.0 - 75.0) | ≤2 | 1(2.6%) | Male | 26 (68.4%) |
|  |  | 2-50 | 13 (34.2%) | Female | 12 (31.6%) |
|  |  | >50 | 24 (63.2%) |  |  |
|  |  |  |  |  |  |
| Other diseases | 56.0 (0.0 - 93.0) | ≤2 | 17 (4.4%) | Male | 248 (63.8%) |
|  |  | 2-50 | 136 (35.0%) | Female | 141 (36.2%) |
|  |  | >50 | 236 (60.7%) |  |  |

General infection: including upper respiratory tract infections, pyelonephritis, and enteric infections.

**Table S2 Comparison of WBC count among different clinical conditions (Stepwise step-down)**

| Diagnosis^c^ | | Median | n | Subset | | |
| --- | --- | --- | --- | --- | --- | --- |
|  |  |  |  | 1 | 2 | 3 |
| Sample^a^ | Hematologic malignancies, Aplastic anemia | 1.855 | 112 | 233.7 |  |  |
|  | Malignant tumors + Common infections | 6.355 | 28 |  | 491.3 |  |
|  | Common infections + Tuberculosis | 7.400 | 58 |  | 521.8 |  |
|  | Tuberculosis | 7.000 | 54 |  | 532.3 | 532.3 |
|  | Organ failure | 8.015 | 64 |  | 580.7 | 580.7 |
|  | Chronic renal diseases | 8.490 | 38 |  | 598.1 | 598.1 |
|  | Malignant tumors | 9.390 | 170 |  | 624.9 | 624.9 |
|  | Other types of diseases | 9.330 | 387 |  |  | 630.5 |
|  | Common infections | 9.710 | 229 |  |  | 642.2 |
|  | Sepsis, septic shock | 12.305 | 16 |  |  | 787.5 |
| Test Statistic | |  |  | .^b^ | 9.763 | 11.985 |
| P value (2-sided test) | |  |  | . | 0.082 | 0.062 |
| Adjusted P value (2-sided test) | |  |  | . | 0.133 | 0.088 |
| Homogeneous subsets are based on asymptotic significances. The significance level is 0.05. | | | | | | |
| a. Each cell shows the sample average rank of diagnosis. | | | | | | |
| b. Unable to compute because the subset contains only one sample. | | | | | | |
| c. Grouping based on the WBC average rank. | | | | | | |

**Table S3 Comparison of neutrophil (NEU%) counts among different clinical conditions (Stepwise step-down)**

| Diagnosis^c^ | | Median | n | Subset | | | |
| --- | --- | --- | --- | --- | --- | --- | --- |
|  |  |  |  | 1 | 2 | 3 | 4 |
| Sample^a^ | Hematologic malignancies, Aplastic anemia | 51.500 | 112 | 367.0 |  |  |  |
|  | Malignant tumors + Common infections | 70.700 | 28 |  | 488.7 |  |  |
|  | Tuberculosis | 72.900 | 54 |  | 528.4 | 528.4 |  |
|  | Chronic renal diseases | 74.850 | 38 |  | 554.4 | 554.4 | 554.4 |
|  | Common infections | 77.300 | 229 |  | 583.4 | 583.4 | 583.4 |
|  | Common infections + Tuberculosis | 75.250 | 58 |  | 584.9 | 584.9 | 584.9 |
|  | Other types of diseases | 76.500 | 387 |  | 588.3 | 588.3 | 588.3 |
|  | Malignant tumors | 79.200 | 170 |  | 649.3 | 649.3 | 649.3 |
|  | Multiple organ failure | 80.050 | 64 |  |  | 651.7 | 651.7 |
|  | Sepsis, septic shock | 83.500 | 16 |  |  |  | 743.7 |
| Test Statistic | |  |  | .^b^ | 11.922 | 11.000 | 11.510 |
| P value (2-sided test) | |  |  | . | 0.064 | 0.088 | 0.074 |
| Adjusted P value (2-sided test) | |  |  | . | 0.090 | 0.124 | 0.104 |
| Homogeneous subsets are based on asymptotic significances. The significance level is 0.05. | | | | | | | |
| a. Each cell shows the sample average rank of diagnosis. | | | | | | | |
| b. Unable to compute because the subset contains only one sample. | | | | | | | |
| c. Grouping based on the NEU% average rank. | | | | | | | |

**Table S4 Culture and IgM detection results of pathogen from different sample types and their association with PCT levels (Mann-Whitney U Test)**

| Microorganism culture | Test results | n | Median | P25–P75 |
| --- | --- | --- | --- | --- |
| Blood culture | Negative | 1151 | 0.640 | 0.185–2.670 |
|  | Positive | 15 | 1.630 | 0.755–3.690 |
|  | Statistics |  | 2.179 | |
|  | P value (two-tailed test) |  | 0.029 | |
| Sputum culture | Negative | 1084 | 0.647 | 0.185–2.670 |
|  | Positive | 82 | 0.681 | 0.216–2.903 |
|  | Statistics |  | 0.525 | |
|  | P value (two-tailed test) |  | 0.599 | |
| Urine/ stool culture | Negative | 964 | 0.620 | 0.180–2.538 |
|  | Positive | 202 | 0.769 | 0.254–3.048 |
|  | Statistics |  | 2.514 | |
|  | P value (two-tailed test) |  | 0.012 | |
| Multiplex-respiratory pathogen IgM detection | Negative | 1145 | 0.650 | 0.187–2.680 |
|  | Positive | 21 | 0.406 | 0.158–2.537 |
|  | Statistics |  | -0.377 | |
|  | P value (two-tailed test) |  | 0.706 | |

**Table S5 Correlation of PCT with CRP, WBC, NEU%, and ESR (Pearson Correlation)**

|  | | PCT | CRP | WBC | NEU% | ESR |
| --- | --- | --- | --- | --- | --- | --- |
| PCT | Pearson Correlation | 1 | 0.220^**^ | 0.185^**^ | 0.098^**^ | 0.024 |
|  | P value (2-tailed test) |  | 0.000 | 0.000 | 0.001 | 0.753 |
|  | n | 1079 | 844 | 1073 | 1065 | 172 |
| CRP | Pearson Correlation | 0.220^**^ | 1 | 0.165^**^ | 0.189^**^ | 0.226^**^ |
|  | P value (2-tailed test) | 0.000 |  | 0.000 | 0.000 | 0.004 |
|  | n | 844 | 862 | 861 | 853 | 162 |
| WBC | Pearson Correlation | 0.185^**^ | 0.165^**^ | 1 | 0.194^**^ | 0.051 |
|  | P value (2-tailed test) | 0.000 | 0.000 |  | 0.000 | 0.488 |
|  | n | 1073 | 861 | 1156 | 1148 | 189 |
| NEU% | Pearson Correlation | 0.098^**^ | 0.189^**^ | 0.194^**^ | 1 | 0.023 |
|  | P value (2-tailed test) | 0.001 | 0.000 | 0.000 |  | 0.758 |
|  | n | 1065 | 853 | 1148 | 1148 | 189 |
| ESR | Pearson Correlation | 0.024 | 0.226^**^ | 0.051 | 0.023 | 1 |
|  | P value (2-tailed test) | 0.753 | 0.004 | 0.488 | 0.758 |  |
|  | n | 172 | 162 | 189 | 189 | 190 |
| **: Correlation is significant at the 0.01 level (2-tailed test). | | | | | | |
| PCT < 0.05 or > 100 were not included in the analysis. | | | | | | |
